# Turning urban wildlife mortality into a surveillance tool: Detection of vector-borne pathogens in carcasses of hedgehogs, squirrels, and blackbirds

**DOI:** 10.1016/j.onehlt.2026.101328

**Published:** 2026-01-12

**Authors:** Karolina Volfová, Václav Hönig, Michal Houda, Petr Papežík, Paulina Maria Lesiczka, Manoj Fonville, Hein Sprong, Barbora Černá Bolfíková, Pavel Hulva, Daniel Růžek, Lada Hofmannová, Jan Votýpka, David Modrý

**Affiliations:** a Department of Parasitology, Faculty of Science, Charles University, Vinicna 7, 128 00 Prague, Czech Republic; b Institute of Parasitology, Biology Centre, Czech Academy of Sciences, Branisovska 31, 370 05 Ceske Budejovice, Czech Republic; c Veterinary Research Institute, Hudcova 70, 621 00 Brno, Czech Republic; d Department of Data Science and Computing Systems, Faculty of Agriculture and Technology, University of South Bohemia, Studentska 1668, 370 05 Ceske Budejovice, Czech Republic; e Department of Zoology, Faculty of Natural Sciences, Comenius University in Bratislava, Ilkovicova 6, 842 15 Bratislava, Slovakia; f Centre for Monitoring of Vectors, Netherlands Institute for Vectors, Invasive plants and Plant health, Netherlands Food and Consumer Product Safety Authority, Geertjesweg 15, 6706, EA, Wageningen, the Netherlands; g Centre for Zoonoses & Environmental Microbiology, Centre for Infectious Disease Control, National Institute for Public Health and the Environment, Antonie van Leeuwenhoeklaan 9, 3721, MA, Bilthoven, the Netherlands; h Faculty of Tropical AgriSciences, Czech University of Life Sciences Prague, Kamycka 129, 165 00 Prague, Czech Republic; i Department of Zoology, Faculty of Science, Charles University, Vinicna 7, 128 00 Prague, Czech Republic; j University of Ostrava, Dvorakova 7, 701 03 Ostrava, Czech Republic; k Department of Botany and Zoology, Faculty of Science, Masaryk University, Kotlarska 2, 611 37 Brno, Czech Republic; l State Veterinary Institute Prague, Sidlistni 24, 165 03 Prague, Czech Republic; m Department of Veterinary Sciences, Faculty of Agrobiology, Food and Natural Resources, Czech University of Life Sciences Prague, Kamycka 129, 165 00 Prague, Czech Republic

**Keywords:** Urban wildlife, Vector-borne pathogens surveillance, Carcasses multi-tissue sampling, *Erinaceus europaeus*, *Erinaceus roumanicus*, *Sciurus vulgaris*, *Turdus merula*

## Abstract

Tick-borne zoonoses pose a major challenge to human and animal health, driving efforts to monitor the distribution, intensity, and diversity of their causative agents. Within the One Health framework, which links human, animal, and environmental health, integrated surveillance strategies are increasingly needed. However, most studies focus on tick vectors, while vertebrate reservoirs are often overlooked due to labour-intensive sampling, the need for specialized skills, and legislative or species protection constraints.

This study evaluated whether carcasses of accidentally killed wildlife (primarily roadkill) can serve as a source of biological material for vector-borne pathogen surveillance, with a focus on urban habitats due to their public health relevance. Hedgehogs, squirrels, and blackbirds were selected as synanthropic species that thrive in cities, are commonly infested by ticks, and act as hosts for zoonotic tick-borne pathogens (TBPs).

A total of 268 carcasses (125 hedgehogs, 55 squirrels, and 88 blackbirds) were collected across multiple Czech cities with public assistance. Overall, 1836 tissue samples were analyzed using multiplex real-time PCR assays targeting over ten microorganisms. Detection efficiency was compared across tissues, with ear and skin consistently the most reliable and versatile sample types. Individual pathogen-host-tissue combinations reached 65–93% efficiency, highlighting the value of multi-tissue sampling. The most prevalent TBPs detected were *Anaplasma phagocytophilum*, *Borrelia burgdorferi* s.l., *Bartonella* spp., and *Rickettsia helvetica*.

In conclusion, carcasses of accidentally killed urban wildlife provide a practical and valuable resource for TBP surveillance, complementing vector-focused methods. This approach supports One Health principles by integrating wildlife monitoring into urban disease surveillance efforts.

## Highlights

• Urban wildlife carcasses enable host-based surveillance of tick-borne pathogens.
• Hedgehogs, squirrels, and blackbirds serve as sentinels for urban TBPs circulation.
• *Anaplasma*, *Borrelia*, *Bartonella*, and *Rickettsia* were the most prevalent pathogens.
• Ear and skin tissues are most reliable; multi-tissue sampling boosts detection.
• Pathogen detection is robust across cadaver autolysis grades, supporting surveillance.

## 1 Introduction

Ticks are recognized vectors of numerous pathogens affecting both humans and animals. Tick-borne diseases, including Lyme borreliosis, tick-borne encephalitis (TBE), babesiosis, and anaplasmosis, are among the most significant vector-borne diseases in the temperate climate zone. Historically, the circulation of tick-borne pathogens (TBPs) was thought to be restricted mainly to natural habitats such as deciduous and mixed forests. In recent decades, reports of medically important tick species and their associated pathogens in urban and peri-urban environments across Europe and beyond have multiplied — a trend that likely reflects both a real ecological expansion and a marked increase in scientific interest and surveillance efforts, as evidenced by the steep rise in published studies on ticks in urban green spaces over the last two decades [1,2]. The circulation of TBPs within city landscapes elevates the risk of human exposure. Urban habitats, characterized by increased fragmentation, reduced biodiversity, and altered host availability which may lead to a higher rate of parasite overdispersion, can significantly influence TBPs dynamics, possibly resulting in even higher pathogen prevalence rates compared to natural habitats [1,3,4].

Numerous studies have documented the presence of key tick vectors, such as *Ixodes ricinus* and *Dermacentor reticulatus*, and the pathogens they transmit in urban environments [[3], [4], [5], [6], [7], [8]]. Despite this, the role of vertebrate hosts, essential for tick feeding and as potential pathogen reservoirs, has received comparatively less attention [[9], [10], [11], [12], [13]], probably due to challenges associated with their sampling—such as labour-intensive procedures, the need for specialized skills, and animal welfare or species protection constraints. To address this gap, our study focuses on four synanthropic species commonly found in urban environments and frequently parasitized by ticks: the European hedgehog (*Erinaceus europaeus*), the Northern white-breasted hedgehog (*E. roumanicus*), the Eurasian red squirrel (*Sciurus vulgaris*), and the Common blackbird (*Turdus merula*). These species, representing insectivores, rodents, and passerine birds, have been identified as competent or probable reservoirs for multiple TBPs [9,[14], [15], [16], [17], [18], [19], [20], [21], [22]]. 

Our methodology relies on the analysis of cadavers, collected predominantly in urban areas. This approach allows for direct detection of TBPs within host tissues, independent of tick presence, providing a more accurate assessment of pathogen circulation in urban ecosystems [11]. Although this study primarily targets TBPs, a comprehensive molecular screening was applied that also encompassed major human pathogens traditionally associated with other arthropod vectors in the region (including *Bartonella* spp., *Francisella tularensis*, and flaviviruses), as ticks are confirmed or putative contributors to their transmission cycles and flaviviruses were screened in a vector-independent manner. From a One Health perspective, the zoonotic relevance of selected pathogens included in the screening—representing those of highest medical relevance in the Czech Republic—is summarized in Supplementary Table 1. Some data presented in this article—particularly concerning the presence of *Borrelia* spp., *Anaplasma phagocytophilum*, *Bartonella* spp., flaviviruses, and *Hepatozoon* spp.—have been previously partially published in separate papers focusing on taxonomic classification of the pathogens, tissue tropism, or genetic diversity in individual host species [[23], [24], [25], [26], [27]]. In contrast, this study offers a broader synthesis, combining all available data to assess co-infections, host age, sex, carcass condition, and habitat type of cadaver origin. This integrative approach enables a more comprehensive view of vector-borne zoonotic pathogen dynamics in urban wildlife and highlights the value of cadaver-based surveillance.

## 2 Methods

### 2.1 Cadaver collection

Cadavers of the target species were primarily collected in urban areas of three major Czech cities: Prague (50.0875° N, 14.4214° E), Brno (49.1925° N, 16.6083° E) and České Budějovice (48.9747° N, 14.4747° E) [coordinates in WGS84, decimal degrees]. A citizen-science approach was employed, whereby members of the public either collected cadavers or reported their locations. Additionally, wildlife rehabilitation centres were contacted and asked to retain relevant cadavers.

Because the degree of decomposition can potentially affect the detectability of pathogen DNA, each cadaver was assigned a decomposition score (autolysis grade) to evaluate the diagnostic efficiency across different decomposition levels. The grading system used in this study is an author-defined classification, inspired by the “degree of degradation” scale described by Szekeres et al. [11]., and adapted to field sampling conditions with an emphasis on early post-mortem changes.

Cadavers were classified into the following categories:

• **Grade 1 A (freshly dead):** Death occurred approximately 1–3 h prior collection in the cold weather period. The carcass is intact, with no odor; fur is firmly attached; the skin is intact with no (or minimal) sloughing, and there is no bloating.
• **Grade 1 B (less fresh):** Death occurred approximately 1–3 h earlier in warm weather or more than 3 h prior collection in cold weather. The carcass is slightly odorous; fur is firmly attached, the skin is intact with no (or minimal) sloughing, early bloating may be present.
• **Grade 2 (moderately decomposed):** Death occurred approximately 4–6 h earlier in warm weather or more than 3–12 h in cold weather. Moderate gas buildup; skin and fur may begin sloughing; odor is clearly noticeable.
• **Grade 3 (advanced decomposition):** The carcass is bloated or beyond the bloated stage, soft, with extensive tissue sloughing and a strong odor.
• **Grade 4 (mummified):** The carcass is flattened and dry, often lacking limbs or internal organs, with minimal odor due to advanced desiccation.

Each cadaver was documented immediately after collection using a standardized collection card, recording the date and time of collection, GPS coordinates, presumed cause of death, autolysis grade, and the finder contact information. Specimens were stored at −20 °C until necropsy.

Based on the GPS coordinates and high-resolution satellite imagery [28], localities were classified into three distinct habitat types:

• **Urban:** Built-up zones (industrial, commercial, public, military, and private units; continuous urban fabric; discontinuous dense urban fabric; discontinuous medium-, low-, or very-low-density urban fabric; green urban areas; sports and leisure facilities).
• **Periurban:** Areas adjacent to urban zones and bordering a rural zone; areas with complex and mixed cultivation patterns.
• **Rural:** Forests, arable land, and other non-urban landscapes.

### 2.2 Cadaver dissection

Cadavers were thawed for 4–8 h (depending on body size and ambient temperature: 8–20 °C) prior to dissection. Dissections followed a previously described protocol [24]. In brief: animals were morphologically identified to species [[29], [30], [31]], sex and age class (juvenile: presence of primary dentice in mammals or juvenile feathers in birds; subadult: sexually immature individuals; and adults), weight and foot length was recorded. For mammals, the following tissues were sampled under sterile conditions: ear, muscle, lungs, blood, liver, spleen, kidneys, urinary bladder, and brain. For blackbird only skin (from the head), muscle, liver, and brain were collected.

Blood coagulum or liquid blood was obtained from the heart or thoracic cavity using a sterile Pasteur pipette. To improve DNA and RNA (for flavivirus detection) preservation, 1 ml of RLT buffer (Qiagen, Hilden, Germany) was added to each sample, which was then stored at −70 °C [32].

Species identity of hedgehogs was further confirmed using a molecular method based on a mitochondrial control region [33,34].

### 2.3 Tissue processing

Tissues were processed as previously described [23,24]. Samples were homogenized (30% *w*/*v*) in RLT buffer (Qiagen, Hilden, Germany) with β-mercaptoethanol, using stainless steel beads in a TissueLyzer II (Qiagen, Hilden, Germany), followed by digestion with proteinase K. After centrifugation, RNA and DNA were extracted from the supernatants using the QIAamp Viral RNA Mini Kit and DNeasy Blood & Tissue Kit (Qiagen, Hilden, Germany) respectively, according to the manufacturer's instructions. All tissues were used for DNA extraction, while only lung, liver, spleen, kidney, and brain samples were used for RNA extraction. Blood for DNA extraction was resuspended in 220 μl of phosphate-buffered saline (PBS); while for RNA extraction [23], it was directly mixed with AVL buffer (Qiagen, Hilden, Germany).

### 2.4 Detection of target microorganisms

All samples were screened for several tick-borne microorganisms using five multiplex real-time PCR (RT-PCR) assays targeting: *Anaplasma phagocytophilum*, *Bartonella* spp., *Neoehrlichia mikurensis*, *Borrelia burgdorferi* s.l., *B. miyamotoi*, *Spiroplasma* spp., *Babesia microti*-like, *Rickettsia helvetica*, and *Francisella* spp.

RT-PCR reactions were performed using iQ Multiplex Powermix with iTaq polymerase (Bio-Rad Laboratories, Hercules, CA, USA) and appropriate primers and probes (see Supplementary Table 2) on a LightCycler 480 (Roche, Basel, Switzerland). The RT-PCR program included: initial activation at 95 °C (5 min), 60 cycles of denaturation (95 °C, 5 s), annealing/extension (60 °C, 35 s), and cooling (37 °C, 20 s). The analysis was performed using second derivative calculations for Cp (crossing point) values. Colour compensation was applied to correct for fluorescence overflow from the dyes used. Amplification curves were visually evaluated in the LightCycler 480 software.

Lungs, liver, spleen, kidney, brain and blood were screened for the presence of flavivirus RNA using a one-step reverse transcription PCR approach, employing universal flavivirus-specific primers and following a protocol described previously [23]. PCR products of expected size were sequenced bidirectionally to confirm the positive detection and to identify the flavivirus species.

Part of the data concerning pathogen genotyping by conventional PCRs and subsequent sequence analyses, specifically *B. burgdorferi* s.l., *Anaplasma phagocytophilum*, *Bartonella* spp., and flaviviruses, were published separately [[23], [24], [25], [26]].

### 2.5 Detection of specific anti-tick-borne encephalitis virus antibodies in live trapped hedgehogs

Altogether 41 hedgehogs were additionally live trapped in urban environments (Prague, Brno, and České Budějovice) and sampled for blood by cardiac puncture. Sera were separated and used for the detection of specific anti-tick-borne encephalitis virus antibodies using a commercially available ELISA kit (Immunozym FSME (TBE) IgG All Species, Progen) according to the manufacturer's instructions.

### 2.6 Statistical analyses

Prevalence rates of the target microorganisms were compared across host age, autolysis grade, and habitat type using Fisher's exact test. For analysis within species groups, we used the asymptotic, permutation based generalized Cochran-Mantel-Haenszel test (CMH), and general independence tests, as described in Agresti [35] and implemented in R via the coin package [36]. The CMH statistic tests for conditional independence in three-way contingency tables in which the third dimension is used as a stratification factor. The general independence test uses no stratification for dependence detection between factors. As all these tests rejected the null hypothesis with considerably low *p*-values, we complemented the analysis with post-hoc Pearson's χ^2^ test of independence (to reveal associations in partial tables), and McNemar's χ^2^ test (to check for marginal homogeneity in partial tables), using Yates's or Edwards's continuity correction, respectively, and Holm-adjusted *p*-values.

## 3 Results

### 3.1 Sample set composition and cadaver characteristics

Altogether, 268 cadavers of the four target species were collected: 42 individuals of *Erinaceus roumanicus* (ER), 83 *E. europaeus* (EE), 55 *Sciurus vulgaris* (SV), and 88 *Turdus merula* (TM). Detailed information for all sampled individuals is provided in Supplementary Table 3.

Both sexes were approximately equally represented across most species, except for blackbirds, in which females slightly predominated (63%, difference statistically not significant, Fisher's exact test). Juvenile individuals constituted roughly one-fifth of the total cadavers, with the lowest proportion of non-adult individuals recorded among blackbirds.

Most cadavers were well-preserved, classified as grade 1 A or 1 B; specimens in advanced stages of autolysis (grade 3) were rare. In a substantial number of cases, the cause of death was not apparent and was therefore recorded as “unknown”. Where the cause could be estimated, road or train collisions were the most common. A notable proportion of blackbirds died from collisions with glass surfaces (Table 1).

**Table 1:** Proportion of cadavers across categorical variables (sex, age class, autolysis grade, and probable cause of death), by species. ER = Northern white-breasted hedgehog (*Erinaceus roumanicus*), EE = European hedgehog (*E. europaeus*), SV = Eurasian red squirrel (*Sciurus vulgaris*), TM = Common blackbird (*Turdus merula*)*.*

Category	ER	EE	SV	TM	total
**Sex**					
**female**	**45%** (19/42)	**45%** (37/83)	**40%** (22/55)	**63%** (55/88)	**50%** (133/268)
**male**	**55%** (23/42)	**51%** (42/83)	**56%** (31/55)	**36%** (32/88)	**48%** (128/268)
**indeterminable**	**0%** (0/42)	**5%** (4/83)	**4%** (2/55)	**1%** (1/88)	**3%** (7/268)
**Age**					
**juvenile**	**21%** (9/42)	**34%** (28/83)	**20%** (11/55)	**7%** (6/88)	**20%** (54/268)
**subadult**	**17%** (7/42)	**19%** (16/83)	**9%** (5/55)	**9%** (8/88)	**13%** (36/268)
**adult**	**62%** (26/42)	**47%** (39/83)	**71%** (39/55)	**84%** (74/88)	**66%** (178/268)
**Autolysis grade**					
**1 A**	**36%** (15/42)	**41%** (34/83)	**73%** (40/55)	**36%** (32/88)	**45%** (121/268)
**1 B**	**40%** (16/42)	**35%** (29/83)	**13%** (7/55)	**31%** (27/88)	**29%** (79/268)
**2**	**21%** (9/42)	**22%** (18/83)	**13%** (7/55)	**33%** (29/88)	**24%** (63/268)
**3**	**5%** (2/42)	**2%** (2/83)	**2%** (1/55)	**0%** (0/88)	**2%** (5/268)
**Probable cause of death**					
**road/train killed**	**38%** (16/42)	**31%** (26/83)	**64%** (35/55)	**27%** (24/88)	**38%** (101/268)
**hit glass**	**0%** (0/42)	**0%** (0/83)	**0%** (0/55)	**13%** (11/88)	**4%** (11/268)
**killed by a predator**	**2%** (1/42)	**5%** (4/83)	**4%** (2/55)	**7%** (6/88)	**5%** (13/268)
**euthanised/died in WRCs**⁎	**0%** (0/42)	**11%** (9/83)	**0%** (0/55)	**0%** (0/88)	**3%** (9/268)
**Usutu virus infection**	**0%** (0/42)	**0%** (0/83)	**0%** (0/55)	**9%** (8/88)	**3%** (8/268)
**pathology not associated with TBPs**	**12%** (5/42)	**1%** (1/83)	**0%** (0/55)	**0%** (0/88)	**2%** (6/268)
**exhaustion/died during hibernation**	**0%** (0/42)	**2%** (2/83)	**0%** (0/55)	**2%** (2/88)	**1%** (4/268)
**trap**	**0%** (0/42)	**0%** (0/83)	**0%** (0/55)	**2%** (2/88)	**1%** (2/268)
**unknown**	**48%** (20/42)	**49%** (41/83)	**33%** (18/55)	**40%** (35/88)	**43%** (114/268)

⁎ Wildlife rehabilitation centres.

### 3.2 Geographic origin of samples

As the main focus of the study was on urban environments, most cadavers originated from major Czech settlements (namely Brno, České Budějovice, and Prague; Supplementary Figure 1). However, several specimens were collected from rural areas, and a substantial portion of samples (not shown in Supplementary Figure 1) were acquired from wildlife rehabilitation centres (WRCs) located in Brno, Jaroměř, Liberec, Pilsen, Prague, and Vlašim.

Cadavers with uncertain collection locations (44/268; 16%) were excluded from habitat-related analyses. The majority of specimens obtained from wildlife rehabilitation centres (WRCs) also lacked precise geographic information (i.e., GPS coordinates). Although WRC staff reported that most animals admitted to their facilities originate from nearby urban or periurban areas, we categorized these cadavers separately to maintain analytical accuracy. In total, precise location data were available for nearly half of the sampled individuals (125/268; 47%) (Table 2).

**Table 2:** Number and proportion of cadavers assigned to each habitat category – urban, periurban, rural, or wildlife rehabilitation centre (WRC). Percentages are calculated relative to the total number of individuals for which habitat classification was available. ER = European hedgehog (*Erinaceus europaeus*), EE = Northern white-breasted hedgehog (*E. roumanicus*), SV = Eurasian red squirrel (*Sciurus vulgaris*), TM = Common blackbird (*Turdus merula*).

	ER		EE		SV		TM		total	
Urban	9	26%	9	12%	14	31%	35	51%	67	30%
**periurban**	2	6%	13	17%	15	33%	17	25%	47	21%
**rural**	0	0%	2	3%	6	13%	3	4%	11	5%
**WRCs**⁎	24	68%	52	68%	10	22%	13	19%	99	44%
**total**	35		76		45		68		224	

⁎ Wildlife rehabilitation centres.

### 3.3 Seasonal patterns of cadaver collection

**Fig. 1:**
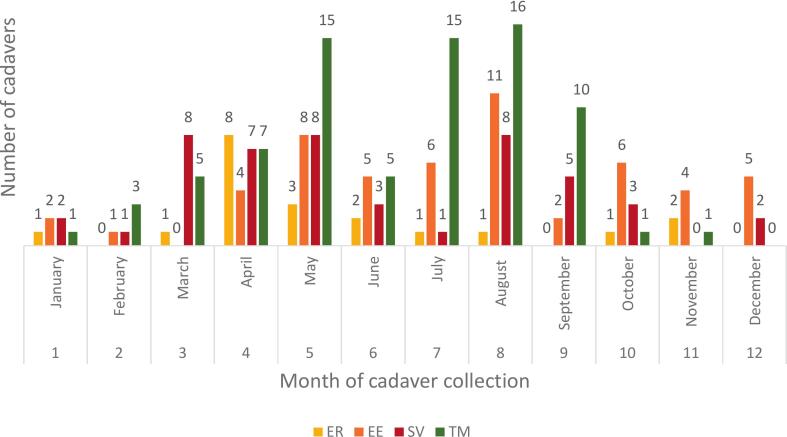
Number of cadavers of the target species collected 2017–2018. ER = European hedgehog (*Erinaceus europaeus*), EE = Northern white-breasted hedgehog (*E. roumanicus*), SV = Eurasian red squirrel (*Sciurus vulgaris*), TM = Common blackbird (*Turdus merula*). (For interpretation of the references to colour in this figure legend, the reader is referred to the web version of this article.)

Regarding seasonal patterns, the majority of cadavers collected for this study were found during spring (April–May) and late summer (July–August) (Fig. 1). However, date-of-death information was unavailable for 73 individuals (27%), mostly because these cadavers have been stored in WRCs for an unspecified period without proper initial documentation (typically when animals died shortly after arrival and prior to receiving any treatment).

### 3.4 Tissue sample availability

Dissection of all cadavers yielded a total of 1836 tissue samples: 324 from *E. roumanicus*, 652 from *E. europaeus*, 448 from *S. vulgaris*, and 412 from *T. merula* (see Supplementary Table 4).

Not all tissue types could be sampled from every individual due to organ loss or advanced tissue degradation. Nonetheless, each of the sampled tissue types was available from at least 83% of cadavers (Table 3).

**Table 3:** Availability of tissue types sampled from the four target species. Tissue unavailability resulted from organ damage or absence (specifically in roadkill), advanced decomposition, or contamination. ER = European hedgehog (*Erinaceus europaeus*), EE = Northern white-breasted hedgehog (*E. roumanicus*), SV = Eurasian red squirrel (*Sciurus vulgaris*), TM = Common blackbird (*Turdus merula*).

Tissue	ear/skin	muscle	blood	lungs#	liver	spleen#	urinary bladder#	kidneys#	brain
**ER**	**100%** (42/42)	**100%** (42/42)	**81%** (34/42)	**69%** (29/42)	**83%** (35/42)	**83%** (35/42)	**95%** (40/42)	**81%** (34/42)	**79%** (33/42)
**EE**	**99%** (82/83)	**99%** (82/83)	**81%** (67/83)	**87%** (72/83)	**83%** (69/83)	**83%** (69/83)	**87%** (72/83)	**88%** (73/83)	**80%** (66/83)
**SV**	**100%** (55/55)	**100%** (55/55)	**89%** (49/55)	**95%** (52/55)	**95%** (52/55)	**91%** (50/55)	**78%** (43/55)	**91%** (50/55)	**76%** (42/55)
**TM**	**99%** (87/88)	**99%** (87/88)	**84%** (74/88)	–	**93%** (82/88)	–	–	–	**93%** (82/88)
**Total**⁎	**99%** (266/268)	**99%** (266/268)	**84%** (224/268)	**85%** (153/180)	**89%** (238/268)	**86%** (154/180)	**86%** (155/180)	**87%** (157/180)	**83%** (223/268)

# indicates tissue types not collected from *Turdus merula*.

⁎ total for all species or total for mammalian species in case tissues not collected from *T. merula.*

### 3.5 Pathogen screening and tissue-specific detection efficiency

All tissue samples obtained from cadavers were screened for the presence of DNA from nine tick-associated microorganisms, most of which are recognized or potential pathogens of humans and/or animals (see Supplementary Table 4).

**Fig. 2:**
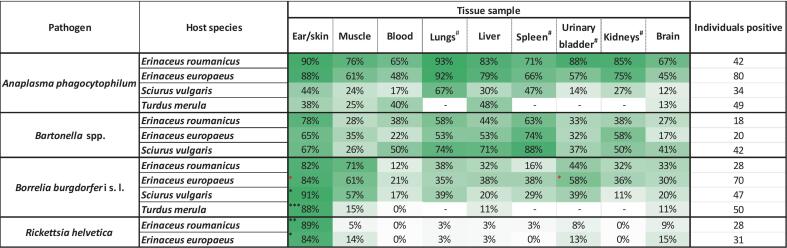
Comparison of tissue-specific detection efficiency for tick-borne pathogens (TBPs) in the four target host species. Detection efficiency was calculated as the percentage of positive detections in a given tissue, relative to the total number of individuals positive for that pathogen in any tissue. Only pathogens with ≥25 positive individuals per host species were included. ^#^indicate tissues not sampled in *Turdus merula*. *indicate statistically significant differences based on McNemar's test with continuity correction and Holm-adjusted *p*-values (*p* < 0.05; ** *p* < 0.01; *** *p* < 0.001). Only tissues with a statistically significantly higher detection rate compared to all other tissues for a given pathogen and host species (i.e. within the same row) are marked. A red asterisk (*) denotes cases where the difference between ear/skin and urinary bladder samples was not statistically significant but those two tissues had a statistically significantly higher detection rate compared to all other tissues.

For each pathogen detected in at least 25 individuals of a given host species, detection efficiency was assessed across different tissue types. It was calculated as the percentage of positive detections in a given tissue relative to all pathogen-positive individuals of that species for which the tissue sample was available (Fig. 2). The ear tissue (or skin, in the case of blackbirds) proved to be the most reliable for detecting the majority of TBP-positive individuals. Overall, ear/skin samples showed a significantly higher prevalence of TBPs compared to all other tissues (McNemar's χ^2^ test with Edwards's continuity correction and Holm adjusted *p*-values; *p* < 0.001) (Fig. 2). Despite this, the highest tissue-specific detection efficiency for individual pathogen–host combinations ranged from 65% to 93%, indicating that the use of multiple tissue types consistently enhanced the sensitivity of pathogen detection.

Across all host species, skin or ear tissue was the most effective sample type for detecting *B. burgdorferi* s.l. and *R. helvetica* (McNemar's tests, *p* < 0.001). In contrast, for *A. phagocytophilum* and *Bartonella* spp., no statistically significant differences in detection efficiency were observed among ear/skin, lung, liver, spleen, and kidney tissues. Similarly, muscle tissue was significantly more effective than other tissues for *B. burgdorferi* detection, with the exception of the urinary bladder, where no significant difference was observed (McNemar's test; *p* < 0.001).

Comparable patterns were also observed at the level of individual host species (Fig. 2). In the case of *A. phagocytophilum*, the pathogen was most consistently detected in lung tissue of mammalian hosts and in the liver of blackbirds (lung was not sampled in *T. merula*). *Borrelia burgdorferi* s.l. and *A. phagocytophilum* were also frequently detected in urinary bladder tissues of both the hedgehog species. *Bartonella* spp. were most reliably detected in spleen samples of squirrels and European hedgehogs, whereas in Northern white-breasted hedgehogs, ear tissue was the most informative one (Fig. 2).

The remaining pathogens were not included in the tissue-specific detection efficiency analysis due to insufficient numbers of positive samples. However, additional pairwise comparisons were conducted and did not reveal any other statistically significant variation in detection rate among tissues.

### 3.6 Pathogen prevalence across host species

**Fig. 3:**
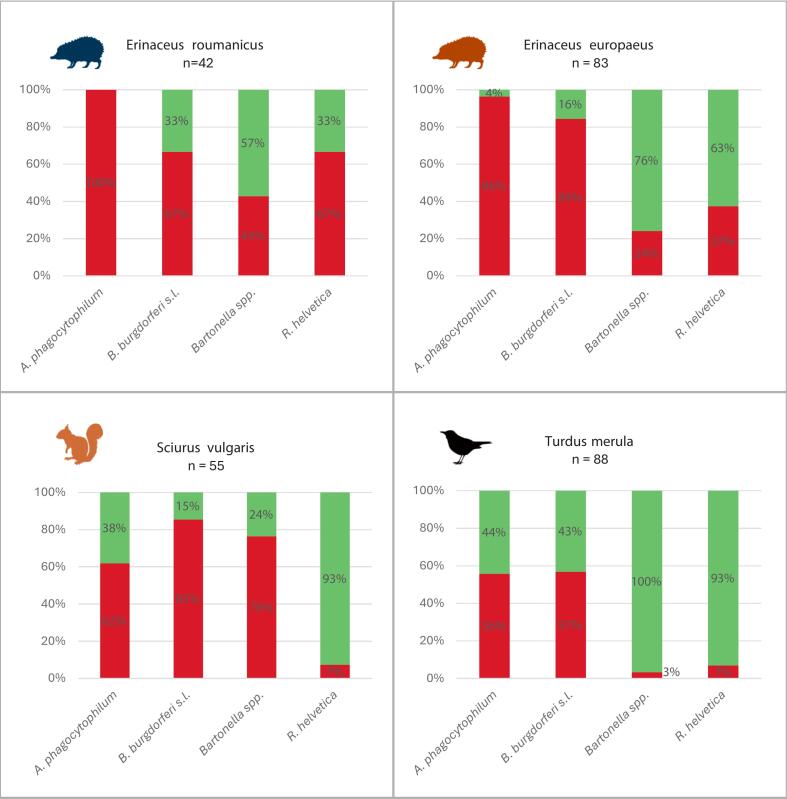
Prevalence of the four most frequently detected pathogens in target host species, based on individual positivity (i.e., individuals positive in at least one tissue sample). Red bars indicate the proportion of positive individuals, while green bars represent negative results. Sample sizes (n) are shown for each host species. (For interpretation of the references to colour in this figure legend, the reader is referred to the web version of this article.)

Out of the nine pathogen groups tested in this study, the four most frequently detected (*A. phagocytophilum*, *B. burgdorferi* s. l., *Bartonella* spp., and *R. helvetica*) are presented in Fig. 3, which shows their prevalence across host species based on individual positivity (i.e., positivity in at least one tissue sample).

Among all tested pathogens, *A. phagocytophilum* was the most prevalent, particularly in both hedgehog species. Almost all individuals of *E. roumanicus* (42/42; 100%) and *E. europaeus* (80/83; 96%) tested positive, indicating a consistently high and statistically significantly higher infection rate in hedgehogs compared to *S. vulgaris* (34/55; 62%) and *T. merula* (49/88; 56%), although the prevalence in these species was still relatively high (Fisher's exact test; *p* < 0.0001). *Borrelia burgdorferi* s.l. showed variable prevalence across host species. The highest, and statistically significantly different, proportions were found in *S. vulgaris* (47/55; 85%) and *E. europaeus* (70/83; 84%), compared to *E. roumanicus* (28/42; 67%) and *T. merula* (50/88; 57%) (Fisher's exact test; *p* < 0.05).

*Bartonella* spp. were most frequently detected in *S. vulgaris* (42/55; 76%), followed by notably lower prevalence in *Erinaceus roumanicus* (18/42; 43%) and *E. europaeus* (20/83; 24%). In contrast, only three specimens of *T. merula* (3/88; 3%) tested positive for *Bartonella*. All differences between the host species were statistically significant (Fisher's exact test; *p* < 0.05).

*Rickettsia helvetica* showed a more heterogeneous distribution across host species. The highest prevalence was detected in *E. roumanicus* (28/42; 67%), followed by *E. europaeus* (31/83; 37%). In contrast, the pathogen was nearly absent in both *S. vulgaris* (4/55; 7%) and *T. merula* (6/88; 7%) (Fisher's exact test; *p* < 0.01).

In addition to the four most prevalent pathogens presented in Fig. 3, five other pathogen taxa were detected at lower frequencies across the studied host species.

*Borrelia miyamotoi* was identified in a total of nine individuals, with positive cases recorded only in mammals: most frequently in *S. vulgaris* (6/55; 11%) and to a lesser extent in *E. europaeus* (3/83; 4%). No positives were detected in *E. roumanicus* or *T. merula*.

*Neoehrlichia mikurensis* was also exclusively found in mammals: in *S. vulgaris* (4/55; 7%) and *E. europaeus* (3/83; 4%); with no positives among *E. roumanicus*.

*Spiroplasma* spp. were detected at low prevalence across three mammalian hosts: *E. europaeus* (4/83; 5%), *E. roumanicus* (2/42; 5%), and *S. vulgaris* (1/55; 2%); no positives were recorded in *T. merula*.

*Francisella* spp. were detected sporadically, with positive cases in *E. europaeus* (2/83; 2%), *S. vulgaris* (1/55; 2%), and *T. merula* (1/88; 1%).

A *Babesia microti*-like DNA was detected only in *E. europaeus* (4/83; 5%), making it the only host species in which this group of protozoan pathogens was detected in this study.

As for the detection of flaviviral RNA, no positive samples were found in either of the two species of hedgehogs, or squirrels. Nevertheless, Usutu virus, a mosquito-borne orthoflavivirus, was detected in blackbirds, as reported previously [23].

From altogether 41 serum samples acquired from hedgehogs live trapped in urban environments (Prague, Brno, and České Budějovice) specific anti-TBEV IgG antibodies were detected in 17 of these individuals, corresponding to a seroprevalence of 41%.

### 3.7 Patterns of co-infections

To investigate patterns of co-infection among the detected TBPs, we conducted a series of asymptotic general independence tests (with no stratification concerning the tissue or the species), and generalized Cochran-Mantel-Haenszel (CMH) tests, with the tissue or the species as the stratification factor. Additionally, the asymptotic general independence test was conducted separately on each species subset; we used a permutation-based implementation of these tests. These tests were all found to be statistically highly significant, with *p* < 2.2 × 10^−16^. On species subsets, these tests were followed by post-hoc pairwise comparisons (whenever possible, that is, where the pathogen was detected at least on one sample) by Pearson's χ^2^ test of independence, with Yate's continuity correction and Holm-adjusted *p*-values. Statistically significant associations (*p* < 0.05 after correction) were considered indicative of non-random co-occurrence.

Among all host species, *S. vulgaris* showed the highest number of statistically significant co-infections. The strongest association was found between *Rickettsia helvetica* and *Neoehrlichia mikurensis* (*p* = 1.6 × 10^−10^), *Rickettsia helvetica* and *Spiroplasma* spp. (*p* = 6.2 × 10^−6^), and *Spiroplasma* spp. and *Neoehrlichia mikurensis* (*p* = 1.0 × 10^−4^).

In *E. europaeus*, three statistically significant co-infections were detected between *Borrelia burgdorferi* s. l. and *Anaplasma phagocytophilum* (*p* = 3.4 × 10^−10^), *Borrelia burgdorferi* s. l. and *Rickettsia helvetica* (*p* = 5.8 × 10^−4^), and *Bartonella* spp. and *Anaplasma phagocytophilum* (*p* = 9.4 × 10^−4^).

In *E. roumanicus*, two statistically significant co-infections were revealed between *Bartonella* spp. and *Anaplasma phagocytophilum* (*p* = 0.0015) and *Borrelia burgdorferi* s. l. and *Rickettsia helvetica* (*p* = 0.0234).

In *T. merula*, only one significant co-infection was observed between *Borrelia burgdorferi* s. l. and *Bartonella* spp. (*p* = 0.0070).

No other associations reached statistical significance.

### 3.8 Effect of cadaver autolysis on pathogen detection

**Fig. 4:**
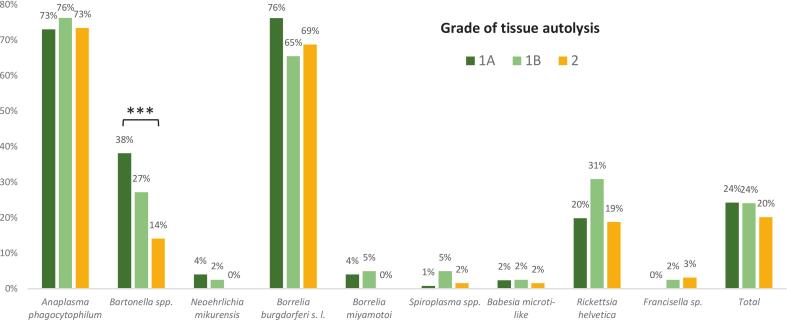
Prevalence of tick-borne pathogens in cadavers of European hedgehog (*Erinaceus europaeus*), Northern white-breasted hedgehog (*E. roumanicus*), Eurasian red squirrel (*Sciurus vulgaris*), and Common blackbird (*Turdus merula*) across different autolysis grades. Grade 1 A corresponds to recently dead individuals with minimal signs of decomposition (∼1–3 h post-mortem in cool weather). Grade 1 B reflects early-stage decomposition (∼1–3 h in warm weather or > 3 h in cool weather). Grade 2 represents moderate decomposition (∼ < 3–6 h post-mortem in warm weather or more than 3–12 h in cool weather). Statistical significance was evaluated using Fisher's exact test; *** indicates *p* < 0.001. (For interpretation of the references to colour in this figure legend, the reader is referred to the web version of this article.)

Prevalence rates of each tested pathogen were compared among cadavers classified into different autolysis grades. Cadavers categorized as grade 3 were excluded from this analysis due to their low overall number. In general, pathogen prevalence was comparable across the different autolysis categories. Only in case of *Bartonella* spp. the prevalence was statistically significantly higher in cadavers with autolysis grade 1 A compared to grade 2 (Fisher's exact test; *p* < 0.001) (Fig. 4).

In a subset of individuals categorized as autolysis grade 3, pathogen detection was still successful. Despite the potentially degraded state of the tissues, at least one pathogen was detected in each of these individuals (see Supplementary Table 5).

### 3.9 Age-related differences in pathogen prevalence

**Fig. 5:**
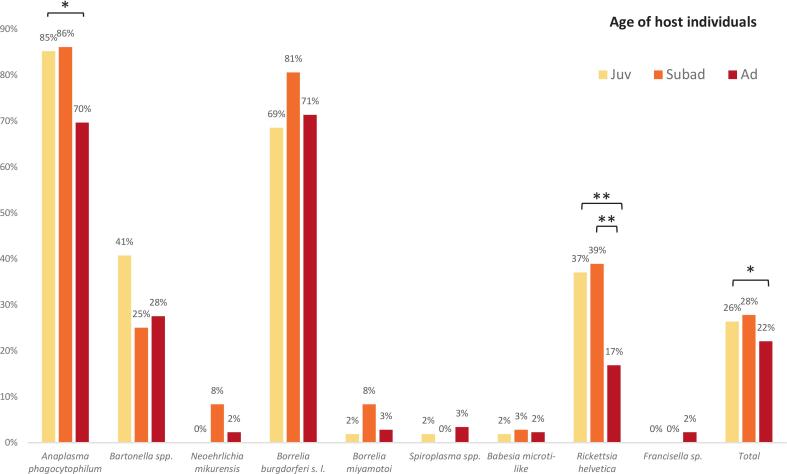
Prevalence of tick-borne pathogens in cadavers of European hedgehog (*Erinaceus europaeus*), Northern white-breasted hedgehog (*E. roumanicus*), Eurasian red squirrel (*Sciurus vulgaris*), and Common blackbird (*Turdus merula*) of different age groups: juvenile (yellow), subadult (orange), adult (red). Statistical significance was tested using Fisher's exact test, * indicates *p* < 0.05, ** indicates *p* < 0.01. (For interpretation of the references to colour in this figure legend, the reader is referred to the web version of this article.)

Despite the overall high prevalence of several TBPs indicating long-term infections, the infection rates were comparable across the three age classes: juvenile, subadult, and adult (Fig. 5). However, certain pathogens exhibited notable differences in age-specific prevalence. *Anaplasma phagocytophilum* showed the highest prevalence in juvenile (85%) and subadult (86%) individuals, with a statistically significant decrease (Fisher's exact test, *p* < 0.01) in adults (70%). Similarly, *R. helvetica* was statistically significantly more frequently detected in juvenile (37%) and subadult (39%) individuals compared to adults (17%) (Fisher's exact test, *p* < 0.01).

For other pathogens, including *Borrelia burgdorferi* s.l., prevalence remained relatively stable across age classes (69–81%), suggesting early and sustained exposure throughout the lifetime of individual animal hosts. *Bartonella* spp. prevalence showed a decreasing trend with age (from 41% in juveniles to 28% in adults), although not statistically significant. Other TBPs such as *Neoehrlichia mikurensis*, *Borrelia miyamotoi*, *Spiroplasma* spp., *Francisella* spp., and piroplasmids were detected at low frequencies and did not show clear age-related patterns.

### 3.10 Habitat-specific differences in pathogen prevalence

**Fig. 6:**
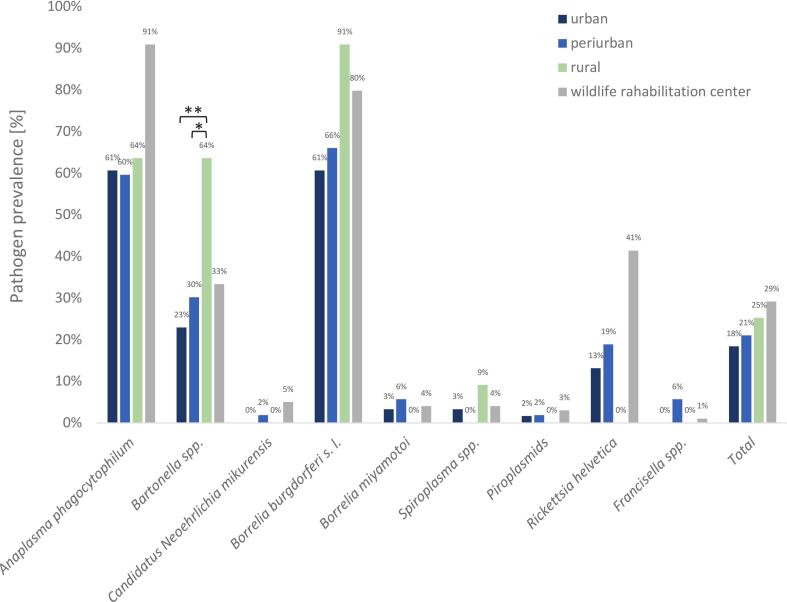
Prevalence of tick-borne pathogens in cadavers of European hedgehog (*Erinaceus europaeus*), Northern white-breasted hedgehog (*E. roumanicus*), Eurasian red squirrel (*Sciurus vulgaris*), and Common blackbird (*Turdus merula*) originating from areas with varying levels of urbanization. The degree of urbanization was classified using high-resolution satellite imagery from the Urban Atlas 2012 [28]: urban (dark blue), periurban (blue), rural (green), wildlife rehabilitation centres (grey). Animals obtained from wildlife rehabilitation centres were typically found in urban or periurban environments and subsequently held in WRC facilities for a variable (often unspecified) period prior to death. Statistical significance was assessed using Fisher's exact test; * indicates *p* < 0.05, ** indicates *p* < 0.01. (For interpretation of the references to colour in this figure legend, the reader is referred to the web version of this article.)

Pathogen prevalence was further evaluated in relation to the habitat category where the cadaver was found (urban, periurban, rural, or WRC; Fig. 6). A statistically significant difference was detected for *Bartonella* spp., with individuals collected in rural areas showing a significantly higher prevalence compared to those from urban (Fisher's exact test; *p* < 0.01), and periurban environments (Fisher's exact test; *p* < 0.05). For other pathogens, no statistically significant differences among habitat categories were observed.

## 4 Discussion

### 4.1 Surveillance approaches and methodological insights

Understanding the transmission cycles of TBPs in both natural and urban environments remains a major challenge. Most studies rely on detecting pathogens in (mostly questing) ticks, making it difficult to infer whether the pathogen originated from the tick or its host [11]. When vertebrate hosts are included, studies most often rely also on sampling engorged ticks removed from animals rather than directly testing host tissues, further limiting insight into the hosts' actual infection status [e.g.,^9^,^37^,^38^,^39^].

Cadaver-based surveillance provides a unique and underutilized method to detect TBPs directly within host tissues, independent of the presence of ticks. Previous studies, such as those by Szekeres et al. [11], demonstrated that TBPs screening in road-killed mammals can reveal reliable insights into urban pathogen circulation. Our study further confirms that this approach can be highly informative, even under moderate to advanced autolysis. Compared to earlier studies focused on the same target vertebrate species cadavers [11,40,41], our results show higher infection rates for most of the tested TBPs. This is likely due to our multi-tissue sampling strategy that allowed detection across various organs, including skin, ear, liver, spleen, and lungs. Tissue tropism varied by pathogen: *B. burgdorferi* s.l. and *R. helvetica* were most efficiently detected in skin and ear samples, while *A. phagocytophilum* and *Bartonella* spp. were more frequently detected in lungs, liver, and spleen, confirming previous findings of pathogen-specific tissue localization [[41], [42], [43], [44], [45], [46], [47], [48], [49]].

Wildlife rehabilitation centres (WRCs) played a key role in the sample acquisition, serving as a valuable source of otherwise inaccessible specimens from urban and periurban areas. However, this strategy introduces several limitations. Animals admitted to WRCs may not be representative of the broader wildlife population; they are often injured, immunocompromised, or more likely to have frequent contact with humans. These factors can skew pathogen prevalence estimates. Furthermore, time spent in captivity may alter infection status (through natural pathogen clearance, progression, or the use of antiparasitics) thereby affecting detectability. The lack of precise geolocation data for many WRC-derived cadavers also limits spatial analyses. Similar concerns have been raised in earlier studies, highlighting the need for cautious interpretation when working with WRC-sourced material [11,50].

### 4.2 Detection reliability and limitations

Importantly, none of the tissue types achieved 100% detection efficiency, which reinforces the importance of multi-tissue sampling for accurate TBPs surveillance. While skin and ear were generally most sensitive, internal organs provided additional value, particularly for detecting pathogens like *A. phagocytophilum* and *Bartonella* spp. The real-time PCR assays employed in our study were further validated by conventional PCRs and sequencing for the three most prevalent pathogens (*A. phagocytophilum*, *B. burgdorferi* s.l., and *Bartonella* spp.), as detailed in earlier publications [[24], [25], [26]]. Although conventional PCR enabled (geno)species and ecotype identification, it was occasionally unsuccessful in samples that tested positive by real-time PCR, leading to lower reported prevalences (especially for *A. phagocytophilum*) or completely negative results (as in the case of blackbirds and *Bartonella* spp.) in the detailed publications.

Pathogen detection remained feasible even in cadavers classified as autolysis grade 3, highlighting the robustness of PCR-based molecular diagnostics under suboptimal tissue preservation. Moreover, for most of the tested TBPs, detection efficiency did not significantly differ across the various autolysis grades. However, *Bartonella* spp. prevalence was significantly higher in fresh cadavers (grade 1 A) compared to moderately decomposed ones (grade 2; *p* < 0.05). This indicates that although decomposition can reduce detection efficiency for some pathogens (particularly those residing in more degradation-prone tissues), valuable molecular data can still be recovered from moderately decomposed specimens.

While pathogen DNA detection confirms exposure, it does not necessarily imply infectiousness or reservoir competence. Particularly for pathogens found in skin or ear samples, it remains unclear whether the presence of DNA reflects active infection or residual DNA of microorganisms that were inoculated into the skin during tick feeding but do not replicate in the vertebrate host. Nevertheless, high prevalence of *B. burgdorferi* s.l. and *R. helvetica* in these tissues and dissemination to internal organs may also indicate replication at the site of inoculation and potential for transmission to co-feeding ticks.

### 4.3 Host-pathogen associations

Our results support the role of synanthropic species (*E. europaeus*, *E. roumanicus*, *S. vulgaris*, and *T. merula*) as valuable sentinels for urban TBPs surveillance.

*Erinaceus roumanicus* showed the highest overall pathogen prevalence, including 100% prevalence for *A. phagocytophilum* and 67% for *B. burgdorferi* s.l., consistent with previous findings in Romanian hedgehogs [51]. While *A. phagocytophilum* and *B. burgdorferi* s.l. prevalence was comparable between *E. roumanicus* and *E. europaeus*, the prevalences of other TBPs suggested species-specific differences in either susceptibility or exposure, as previously hypothesized by Dziemian et al. [52]. The consistently high TBPs prevalences in both hedgehog species underscores their significant role as a reservoir for these pathogens. Supporting this, a study by Jahfari et al. [53] and Springer et al. [54] found that European hedgehogs contribute to the maintenance of various TBPs in urban and suburban areas, highlighting their involvement in enzootic transmission cycles. Additionally, research by Szekeres et al. [11] demonstrated that *E. roumanicus* harbours a diverse range of TBPs, including *B. afzelii* and *R. helvetica*, further reinforcing its epidemiological significance. 

*Sciurus vulgaris* exhibited a high prevalence of *B. burgdorferi* s.l. (85%) and *Bartonella* spp. (76%), consistent with previous findings of these pathogens in Eurasian red squirrels from Belgium [55], Germany [40], and Lithuania [41].

*Turdus merula* showed moderate prevalences of *A. phagocytophilum* and *B. burgdorferi* s.l., but very low prevalence for *Bartonella* spp. Previously proposed host specificity of *Bartonella* spp. to mammals was first challenged by the detection of *Bartonella* DNA in sea turtles [56]. Since then, multiple studies have investigated the presence of *Bartonella* spp. in avian hosts, confirming the detection of *Bartonella* DNA in various bird species (e.g., [[57], [58], [59], [60]]). However, to the best of our knowledge, such detection has not yet been reported in *T. merula*. Further research is needed to clarify the host specificity of *Bartonella* spp. and the potential roles of avian species and their ectoparasites in the ecology and transmission dynamics of these bacteria.

### 4.4 Patterns and implications of co-infection

Co-infections were frequently detected with some associations being statistically significant, especially between *Borrelia burgdorferi* s. l. and other pathogens (two co-infections for *E. europaeus* and one for *E. roumanicus* and *T. merula*), and between *Anaplasma phagocytophilum* and *Bartonella* spp. (in *E. roumanicus* and *E. europaeus*). These findings of tick-borne pathogens co-infections are consistent with the general data reviewed by Gomez-Chamorro et al. [61].

Co-infections can influence disease dynamics in wildlife and complicate diagnosis in both veterinary and human medicine. Their frequent occurrence, especially in urban-adapted mammals, reinforces the importance of using comprehensive diagnostic tools and considering co-infection as a potential confounding factor in disease surveillance and modelling.

### 4.5 Ecological and demographic drivers of pathogen prevalence

Infection patterns also varied by host age and habitat. Juvenile and subadult hosts had significantly higher prevalence of *A. phagocytophilum* and *R. helvetica* than adults. For *B. burgdorferi* s.l. and *Bartonella* spp., prevalence remained relatively stable or showed non-significant trends across age groups. These findings suggest early-life exposure to TBPs and potentially age-dependent susceptibility or immune-mediated clearance.

*Bartonella* spp. prevalence was significantly higher in rural areas, possibly due to differences in host species richness, density of other arthropod vectors (most likely fleas), or land-use patterns. No significant differences were found for other pathogens. However, given the synanthropic habits of hedgehogs, squirrels, and blackbirds, and their frequent exposure to ticks in fragmented urban green spaces, these species serve as valuable sentinels for detecting local TBPs diversity. The presence of zoonotic pathogens such as *B. burgdorferi* s.l., *A. phagocytophilum*, and *Bartonella* spp. in these animals underlines the public health relevance of TBPs monitoring in urban wildlife.

### 4.6 Arboviruses in urban wildlife

To evaluate the presence of flaviviral RNA in the sampled wildlife, we screened all cadaver tissue samples using molecular assays. Usutu virus was the only flavivirus detected, and it was identified exclusively in blackbirds (*T. merula*) [23].

All mammalian cadavers tested negative for flaviviral RNA. Given that hedgehogs are considered probable reservoirs of tick-borne encephalitis virus (TBEV) [14,15,62], a separate serological screening was also performed on live-trapped individuals to assess TBEV exposure. The contact of hedgehogs with TBEV was confirmed as the seroprevalence rate reached 41% in the analyzed samples. These animals were not included in the cadaver pathogen screening dataset, and no further details are therefore provided here for them.

Despite this relatively high antibody prevalence, TBEV RNA was not detected in any of the hedgehog cadavers. Although RNA degradation in post-mortem tissues cannot be entirely excluded, this explanation is unlikely to fully account for the negative results, as Usutu virus RNA was successfully detected in blackbird cadavers processed using the same sampling and extraction protocols.

Rodents and insectivores are generally considered natural reservoirs of TBEV [14,63], but specific data for squirrels and hedgehogs remain scarce. While previous studies have reported high seroprevalence in these species [62,64,65], direct detection of TBEV RNA or successful virus isolation appears to be rare [64,65]. This discrepancy may be due to the short persistence of the virus in tissues and body fluids following infection—unlike in bank and field voles, where longer-term TBEV RNA detection has been reported [[66], [67], [68]]. It is also possible that a rapid, antibody-mediated immune response prevents long-term RNA detectability in hedgehog tissues similar to human sera [69,70].

## 5 Conclusions

This study demonstrates that carcasses of synanthropic vertebrates constitute a valuable, underutilized resource for the surveillance of a broad spectrum of zoonotic vector-borne pathogens in urban areas. Through multi-tissue molecular screening of 268 cadavers across four synanthropic species—*Erinaceus europaeus, E. roumanicus, Sciurus vulgaris*, and *Turdus merula*—we detected high prevalence rates of several key tick-borne pathogens, including *Anaplasma phagocytophilum*, *Borrelia burgdorferi* s.l., *Bartonella* spp., and *Rickettsia helvetica*. Notably, employing multiple tissue types substantially enhanced the overall detection sensitivity, while even moderately or advanced autolyzed specimens retained sufficient diagnostic value. Hedgehogs, particularly *E. roumanicus*, showed consistently high infection rates and extensive co-infections, reinforcing their importance in urban TBPs cycles. Meanwhile, *S. vulgaris* and *T. merula* contributed distinct host-pathogen profiles, including the absence of several tested TBPs in blackbirds and elevated *Bartonella* prevalence in squirrels. Habitat- and age-related variation in pathogen prevalence further underscore the influence of ecological and demographic factors. Together, these findings support the integration of cadaver-based host surveillance into urban zoonotic disease monitoring strategies, offering complementary insights to conventional tick-based approaches and advancing our understanding of TBPs circulation in human-dominated landscapes.

## CRediT authorship contribution statement

**Karolina Volfová:** Writing – review & editing, Writing – original draft, Visualization, Investigation, Formal analysis, Data curation. **Václav Hönig:** Writing – review & editing, Writing – original draft, Visualization, Investigation, Funding acquisition, Formal analysis, Data curation. **Michal Houda:** Writing – review & editing, Methodology, Investigation, Formal analysis. **Petr Papežík:** Investigation. **Paulina Maria Lesiczka:** Investigation. **Manoj Fonville:** Methodology, Investigation. **Hein Sprong:** Supervision, Methodology. **Barbora Černá Bolfíková:** Methodology, Investigation. **Pavel Hulva:** Methodology. **Daniel Růžek:** Supervision, Methodology. **Lada Hofmannová:** Methodology, Investigation, Data curation. **Jan Votýpka:** Writing – review & editing, Supervision, Methodology, Funding acquisition. **David Modrý:** Writing – review & editing, Supervision, Resources, Project administration, Methodology, Funding acquisition.

## Ethics statement

The study was conducted as a citizen-science project and relied exclusively on carcasses of animals found dead in the field or obtained dead from wildlife rehabilitation centres. No live animals were collected, handled, or euthanized specifically for this study. In accordance with Czech animal protection legislation (Act No. 246/1992 Coll. and implementing regulations), research based solely on dead specimens does not require approval by an institutional ethics committee.

## Funding

This research was funded by the 10.13039/501100001824Czech Science Foundation (grant number 17-16009S), by the Ministry of Education, Youth and Sports of the Czech Republic [project no. LUC23151, INTER-EXCELLENCE II]; and the 10.13039/501100003243Ministry of Health of the Czech Republic (grants number NU23–05-00511 and NU23–09-00049). H.S. and M.F. were financially supported by the 10.13039/501100002999Dutch Ministry of Health, Welfare and Sport (VWS) and by a grant from the European Interreg North Sea Region program as part of the NorthTick project.

## Declaration of competing interest

The authors declare that they have no known competing financial interests or personal relationships that could have appeared to influence the work reported in this article.

## Data Availability

The data supporting the findings of this study are available from the corresponding author upon reasonable request.

## Appendix A Supplementary data

Supplementary material 1 Image 1

Supplementary material 2 Image 2

Supplementary material 3 Image 3

Supplementary material 4 Image 4

Supplementary material 5 Image 5

Supplementary material 6 Image 6
