# Supplementary material for: Turning urban wildlife mortality into a surveillance tool: Detection of vector-borne pathogens in carcasses of hedgehogs, squirrels, and blackbirds
Source: One Health. 2026 Jan 12;22:101328. doi: 10.1016/j.onehlt.2026.101328 (PMC12856192; doi:10.1016/j.onehlt.2026.101328)

**Supplementary Figure 1. Map of cadaver origins.** The map includes only individuals for which exact find locations were available and GPS coordinates were recorded.
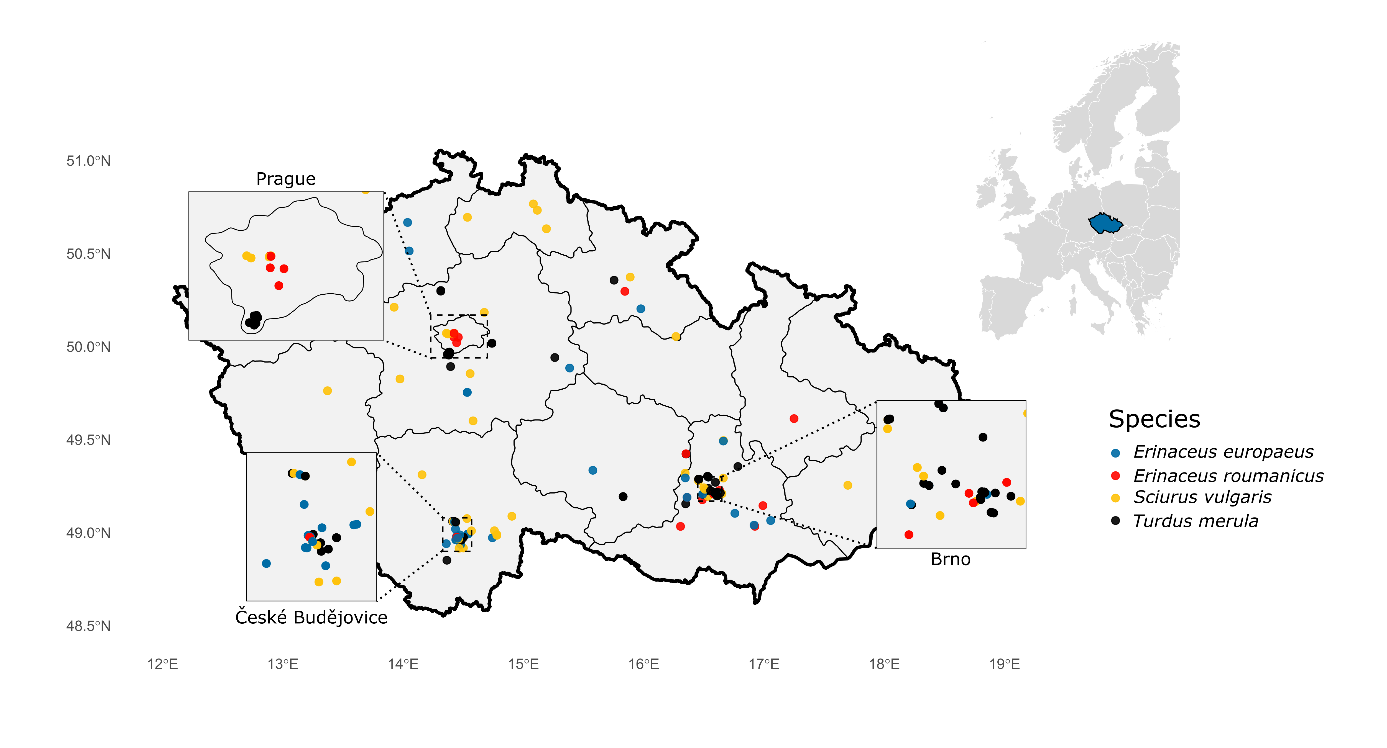

Supplement: Supplementary file 1 — Supplementary material 1 [file mmc1.docx]
