# Supplementary material for: Turning urban wildlife mortality into a surveillance tool: Detection of vector-borne pathogens in carcasses of hedgehogs, squirrels, and blackbirds
Source: One Health. 2026 Jan 12;22:101328. doi: 10.1016/j.onehlt.2026.101328 (PMC12856192; doi:10.1016/j.onehlt.2026.101328)
Supplement: Supplementary file 3 — Supplementary material 3 [file mmc3.docx]

**Supplementary Table 2.** Primers and probes and their combinations used for the multiplex real-time PCR detection of the target tick-associated microorganisms in the tissue samples of cadavers of European hedgehogs (*Erinaceus europaeus*), Northern white-breasted hedgehogs (*E. roumanicus*), Eurasian red squirrel (*Sciurus vulgaris*), and Common blackbirds (*Turdus merula*).

| **Mastermix I** | | | | |  |
| --- | --- | --- | --- | --- | --- |
| **Microorganism** | **Gene** | **Oligonucleotide** | **Sequence** | **Reference** |  |
| *B. burgdorferi* s.l. | *OspA* | Forward primer | 5'-AAT ATT TAT TGG GAA TAG GTC TAA-3' | Heylen, D.J.A.; Tijsse, E.; Fonville, M.; Matthysen, E.; Sprong, H. Transmission dynamics of *Borrelia burgdorferi* s.l. in a bird tick community. Environ. Microbiol 2013, 15, 663–673. |  |
|  |  | Reverse primer | 5'-CTT TGT CTT TTT CTT TRC TTA CA-3' |  |  |
|  |  | Probe | 5'-**Atto520**-AAG CAA AAT GTT AGC AGC CTT GA-**BHQ1**-3' |  |  |
|  |  |  |  |  |  |
|  | *FlaB* | Forward primer | 5'-CAG AIA GAG GTT CTA TAC AIA TTG AIA TAG A-3' |  |  |
|  |  | Reverse primer 1 | 5'-GTG CAT TTG GTT AIA TTG CGC-3' |  |  |
|  |  |  |  |  |  |
|  |  | Reverse primer 2 | 5'-GTG CAT TTG GTT AIA TTG TGC-3' |  |  |
|  |  |  |  |  |  |
|  |  | Probe | 5'-**Atto425**-CAA CTI ACA GAI GAA A **BHQ-1**-dTT AAI AGA ATT GCT GAI CA-**Pho-3**' |  |  |
|  |  |  |  |  |  |
| *B. myiamotoi* | *FlaB* | Forward primer | 5'-AGA AGG TGC TCA AGC AG-3' | Hovius, J.W.R.; de Wever, B.; Sohne, M.; Brouwer, M.C.; Coumou, J.; Wagemakers, A.; Oei, A.; Knol, H.; Narasimhan, S.; Hodiamont, C.J.; Jahfari, S.; Pals, S.T.; Horlings, H.M.; Fikrig, E.; Sprong, H.; van Oers, M.H.J. A case of meningoencephalitis by the relapsing fever spirochaete *Borrelia miyamotoi* in Europe. Lancet 2013, 382, 658 |  |
|  |  | Reverse primer | 5'-TCG ATC TTT GAA AGT GAC ATA T-3' |  |  |
|  |  | Probe | 5'-**ATTO647N**-AGC ACA ACA GGA GGG AGT TCA AGC-**BHQ2**-3' |  |  |
|  |  |  |  |  |  |
| **Mastermix II** | | | | |  |
| *Anaplasma phagocytophilum* | *MSP 2* | Forward primer | ATG GAA GGT AGT GTT GGT TAT GGT ATT | Courtney, J.W.; Kostelnik, L.M.; Zeidner, N.S.; Massung, R.F. Multiplex real-time PCR for detection of *Anaplasma phagocytophilum* and *Borrelia burgdorferi*. J Clin Microbiol. 2004 Jul;42(7):3164-8. doi: 10.1128/JCM.42.7.3164-3168.2004. PMID: 15243077; PMCID: PMC446246. |  |
|  |  | Reverse primer | TTG GTC TTG AAG CGC TCG TA |  |  |
|  |  | Probe | 5'-**Atto425 -**TGG TGC CAG GGT TGA GCT TGA GAT TG-**BHQ1**-3' |  |  |
|  |  |  |  |  |  |
| *Neoehrlichia mikurensis* | GroEl | Forward primer | CCT TGA AAA TAT AGC AAG ATC AGG TAG | Jahfari, S.; Fonville, M.; Hengeveld, P. et al. Prevalence of *Neoehrlichia mikurensis* in ticks and rodents from North-west Europe. Parasites Vectors 5, 74 (2012). https://doi.org/10.1186/1756-3305-5-74 |  |
|  |  | Reverse primer | CCA CCA CGT AAC TTA TTT AGC ACT AAA G |  |  |
|  |  | Probe | 5'-**ATTO520**-CCT CTA CTA ATT ATT GCX GAA GAT GTA GAA GGT GAA GC- **Pho**-3' |  |  |
|  |  |  |  |  |  |
| *Babesia microti -*like | 18S rRNA | Forward primer | CAG CTT GAC GGT AGG GTA TTG G | Radzijevskaja J., Paulauskas A., Rosef O. Prevalence of *Anaplasma phagocytophilum* and *Babesia divergens* in *Ixodes* *ricinus* ticks from Lithuania and Norway. International Journal of Medical Microbiology 298 (2008) S1, 218-221. https://doi.org/10.1016/j.ijmm.2008.01.008 |  |
|  |  | Reverse primer | TCG AAC CCT AAT TCC CCG TTA |  |  |
|  |  | Probe | 5'-**ATTO647N**-CGA GGC AGC AAC GG- **BHQ2**-3' |  |  |
| **Mastermix III** | | | | |  |
| *Spiroplasma* spp*.* | *RpoB* | Forward primer | 5'-TGT TGG ACC AAA CGA AGT TG-3' | Subramanian, G.; Sekeyova, Z.; Raoult, D.; Mediannikov, O. Multiple tick-associated bacteria in *Ixodes ricinus* from Slovakia. Ticks Tick Borne Dis. 2012, 3, 406. |  |
|  |  | Reverse primer | 5'-CCA ACA ATT GGT GTT TGG GG-3' |  |  |
|  |  | Probe | 5'-**Atto425**-GCT AAC CGT GCT TTA ATG GG-**BHQ1**-3' |  |  |
| *B. microti* | *ITS* | Forward primer | 5'-CTC ACA CAA CGA TGA AGG ACG CA-3' | Kazimirova, M.; Hamsikova, Z.; Spitalska, E.; Minichova, L.; Mahrikova, L.; Caban, R.; Sprong, H.; Fonville, M.; Schnittger, L.; Kocianová, E. Diverse tick-borne microorganisms identified in free-living ungulates in Slovakia. Parasites Vectors 2018, 11, 495. |  |
|  |  | Reverse primer | 5'-AAC AGA GGC AGT GTG TAC AAT ACA TTC AGA-3' |  |  |
|  |  | Probe | 5'-**Atto520**-GCA +GAA TTT AG+C AAA T+CA ACA GG**-BHQ1**-3' |  |  |
|  |  |  |  |  |  |
| *R. helvetica* | *gltA* | Forward primer | 5'-ATG ATC CGT TTA GGT TAA TAG GCT TCG GTC-3' | de Bruin, A.; van Leeuwen, A.D.; Jahfari, S.; Takken, W.; Földvári, M.; Dremmel, L.; Sprong, H.; Földvári, G. Vertical transmission of *Bartonella schoenbuchensis* in *Lipoptena cervi*. Parasit Vectors. 2015 Mar 21; 8:176. doi: 10.1186/s13071-015-0764-y. PMID: 25889985; PMCID: PMC4374187. |  |
|  |  | Reverse primer | 5'-TTG TAA GAG CGG ATT GTT TTC TAG CTG TC-3' |  |  |
|  |  | Probe | 5'**-Atto647N**-CGA TC+ C+A CG+ TG+ CCG CAG T-**BHQ2**-3' |  |  |
|  |  |  |  |  |  |
| **Mastermix IV** | | | | |  |
| *Francisella* spp. | *IS111 1* | Forward primer | 5'-CAA GCA ATT GGT AGA TCA GTT GG-3' | Janse, I.; Hamidjaja, R.A.; Bok, J.M.; van Rotterdam, B.J. Reliable detection of *Bacillus anthracis*, *Francisella tularensis* and *Yersinia pestis* by using multiplex qPCR including internal controls for nucleic acid extraction and amplification. BMC Microbiol. 2010 Dec 8; 10:314. doi: 10.1186/1471-2180-10-314. |  |
|  |  | Reverse primer | 5'-GAC AAC AAT ATT TCT ATT GGA TTA CCT AAA-3' |  |  |
|  |  | Probe | 5'-**ATTO647N**-ACC ACT AAA ATC CAT GCT ATG ACT GAT G-**BHQ1**-3' |  |  |
|  |  |  |  |  |  |
| *F. tularensis* | *fopA* | Forward primer | 5'-GCG CTT TGA CTA ACA AGG ACA-3' |  |  |
|  |  | Reverse primer | 5'-CCA GCA CCT GAT GGA GAG TT-3' |  |  |
|  |  | Probe | 5'-**Atto425-**TGG CCA GTG GTA CTT AGG TGT AGA TGC TA-**BHQ1**-3' |  |  |
|  |  |  |  |  |  |
| **Mastermix V** | | | | |  |
| *Bartonella* sp. | *ssrA* | Forward primer | 5'-GCT ATG GTA ATA AAT GGA CAA TGA AAT AA-3' | Diaz, M.H.; Bai, Y.; Malania, L.; Winchell, J.M.; Kosoy, M.Y. Development of a novel genus-specific real-time PCR assay for detection and differentiation of *Bartonella* species and genotypes. J. Clin. Microbiol. 2012, 50, 1645–1649. |  |
|  |  | Reverse primer | 5'-GCT TCT GTT GCC AGG TG-3' |  |  |
|  |  | Probe | 5'-**ATTO520**-ACC CCG CTT AAA CCT GCG ACG-**BHQ1**-3' |  |  |
