# Supplementary material for: Turning urban wildlife mortality into a surveillance tool: Detection of vector-borne pathogens in carcasses of hedgehogs, squirrels, and blackbirds
Source: One Health. 2026 Jan 12;22:101328. doi: 10.1016/j.onehlt.2026.101328 (PMC12856192; doi:10.1016/j.onehlt.2026.101328)
Supplement: Supplementary file 6 — Supplementary material 6 [file mmc6.docx]

**Supplementary Table 5.** Detection of tick-borne pathogens in individuals categorized as autolysis grade 3. At least one of the nine tested pathogens was successfully detected in each individual. Host species include *Erinaceus roumanicus* (ER), *Erinaceus europaeus* (EE), and *Sciurus vulgaris* (SV). "+" indicates a positive result, "-" indicates a negative result.

|  | ***A. phagocytophilum*** | ***Bartonella* spp.** | ***B. burgdorferi* s. l.** | ***Spiroplasma* spp.** | ***R. helvetica*** |
| --- | --- | --- | --- | --- | --- |
| ER | **+** | - | - | - | **+** |
| ER | **+** | - | **+** | - | **+** |
| EE | **+** | **+** | **+** | - | - |
| EE | - | - | - | **+** | - |
| SV | - | - | **+** | - | - |
